# Beyond visible: giant bulk photovoltaic effect for broadband neuromodulation

**DOI:** 10.1038/s41377-024-01697-7

**Published:** 2025-01-03

**Authors:** Xueping Chai, Zhipei Sun

**Affiliations:** 1 College of Mechanical and Electronic Engineering, Tarim University, Hongqiao South Rd. 705, Aral, 843300 China; 2 QTF Centre of Excellence, Department of Electronics and Nanoengineering, Aalto University, Tietotie 3, FI-02150 Espoo, Finland

**Keywords:** Optical techniques, Photonic devices

## Abstract

The giant bulk photovoltaic effect in tellurene nanomaterials has been harnessed to enable broadband infrared neuromodulation, expanding the potential for safe, non-invasive neural stimulation and highlighting the importance of material innovation in advancing infrared photonic applications.

Efficient conversion of light into electricity is crucial across a wide range of fields, from imaging and biological sensing to clean energy and free-space communication. The bulk photovoltaic effect (BPVE) has recently attracted attention for its remarkable ability to surpass the traditional efficiency limits of conventional solar cells, known as the Shockley-Queisser limit^1^. Unlike typical solar cells, which rely on p-n junctions to generate electricity, BPVE operates in materials without a center of symmetry (non-centrosymmetric materials)^2^, producing a photocurrent directly from light exposure without the need for a p-n junction. This unique mechanism offers exciting potential for more efficient light-to-electricity conversion in cutting-edge applications.

In the late 20th century, scientists began focusing on identifying materials suitable for the BPVE and understanding its underlying mechanisms, as illustrated in Fig. 1. Early studies revealed that certain materials, such as ferroelectrics like BaTiO_3_ and LiNbO_3_, along with specific organic compounds, showed promising BPVE properties^3^. These initial discoveries laid the groundwork for optimizing these materials to improve their performance. As material science advanced, researchers discovered new BPVE-active materials, including organic semiconductors and two-dimensional materials^4,5^, which greatly expanded BPVE applications by achieving impressive efficiencies in the ultraviolet (UV) and visible (VIS) light ranges^6^.

**Fig. 1:**
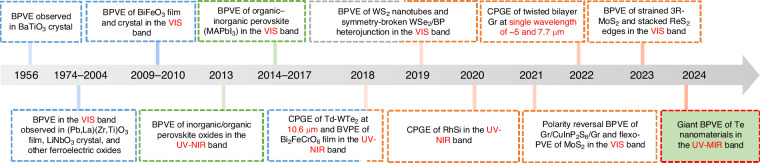
The timeline of selected key developments in the BPVE study^2,4,6,8,11–22^. NIR near-infrared, MIR mid-infrared

Currently, however, BPVE responses are generally limited to a narrow range of wavelengths due to interband optical transitions in conventional semiconductors and heterostructures. While some phenomena (e.g., Berry curvature and scattering in semimetals) could potentially support BPVE generation in the infrared range, achieving photoelectric conversion with polarized single-wavelength light remains challenging^7^. This effect, related to the circular photogalvanic effect, highlights the need for further research into materials that can achieve a broadband BPVE response, especially in the infrared range, where detection remains challenging but holds immense potential for applications.

Recently, a collaborative research team led by Professor Weida Hu from the Shanghai Institute of Technical Physics, Chinese Academy of Sciences, achieved a major breakthrough in extending BPVE into the MIR range^8^. Building on their prior work with low-dimensional tellurene (Te) nanomaterials^9,10^, the team synthesized Te nanomaterials using chemical vapor deposition, resulting in devices that exhibit a wide-ranging BPVE response—from UV to MIR wavelengths, covering ~0.39 to 3.8 µm. Using scanning photocurrent mapping, the researchers evaluated the infrared response of these materials, finding it to be consistently robust across the VIS to infrared spectrum and primarily localized within the channel itself. Remarkably, the Te materials demonstrated a high photocurrent density of ~70 A cm^−2^ under infrared light, greatly surpassing the performance of other known BPVE materials.

Exploring potential applications, the team tested Te nanomaterials for broadband optical neuromodulation. When co-cultured with primary cortical neurons, the Te nanoflakes successfully triggered action potentials (electrical impulses) in neurons when exposed to light at various wavelengths, including ~637, 940, 1310, and 1550 nm, as shown in Fig. 2. This effect was achieved without any extra external electrical stimulation, yet the action potentials produced were comparable to those seen with traditional electrical methods. The process occurs as photogenerated carriers from the BPVE diffuse into the extracellular medium, altering the transmembrane voltage and activating neuronal activity. Importantly, the Te nanoflakes showed no signs of cytotoxicity or cell damage, even after prolonged exposure, underscoring their biocompatibility and suitability for potential biomedical applications.

**Fig. 2:**
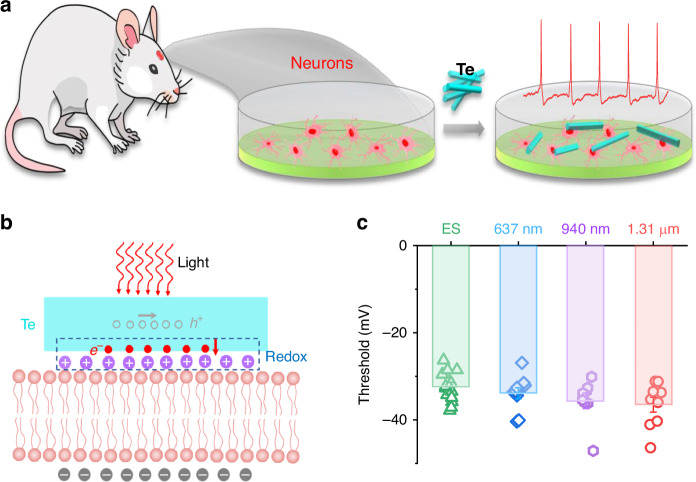
Optical neuromodulation using Te based nanomaterials with BPVE. **a** Schematic of the co-culture setup for mouse primary cortical neurons with Te nanomaterials. Red shapes represent the mouse cortical neurons, and cyan shapes indicate the Te nanomaterials. **b** Illustration of the broadband neuromodulation mechanism facilitated by the interaction between neurons and Te nanomaterials. A redox process occurs at the interface, with purple and gray spheres indicating extracellular cations and intracellular anions, respectively, while red and yellow hollow spheres represent photogenerated electrons and holes. **c** Action potential threshold generated by primary cortical neurons co-cultured with Te nanomaterials at different wavelengths, demonstrating the neuromodulation effect. The figure is provided courtesy of Prof. Weida Hu

These findings highlight the strong potential of Te for broadband infrared applications, particularly in the field of non-invasive neuromodulation, where it could offer safer, more versatile options for influencing neural activity. Further research into Te and similar nanomaterials is crucial, as advancements in this area could open new pathways for safer medical technologies and expand the possibilities of infrared applications across various fields, from biomedical engineering to advanced imaging.

## References

[1] Spanier, J. E. et al. Power conversion efficiency exceeding the shockley–queisser limit in a ferroelectric insulator. *Nat. Photonics* **10**, 611–616 10.1038/nphoton.2016.143 (2016).

[2] Akamatsu, T. et al. A van der Waals interface that creates in-plane polarization and a spontaneous photovoltaic effect. *Science* **372**, 68–72 10.1126/science.aaz9146 (2021). 33795452 10.1126/science.aaz9146

[3] Glass, A. M., Von Der Linde, D. & Negran, T. J. High‐voltage bulk photovoltaic effect and the photorefractive process in LiNbO_3_. *Appl. Phys. Lett.* **25**, 233–235 10.1063/1.1655453 (1974).

[4] Liang, Z. H. et al. Strong bulk photovoltaic effect in engineered edge-embedded van der Waals structures. *Nat. Commun.* **14**, 4230 10.1038/s41467-023-39995-0 (2023). 37454221 10.1038/s41467-023-39995-0 PMC10349808

[5] Dang, Y. Y. & Tao, X. T. Recent progress of bulk photovoltaic effect in acentric single crystals and optoelectronic devices. *Matter* **5**, 2659–2684 10.1016/j.matt.2022.06.011 (2022).

[6] Dong, Y. et al. Giant bulk piezophotovoltaic effect in 3R-MoS_2_. *Nat. Nanotechnol.* **18**, 36–41 10.1038/s41565-022-01252-8 (2023). 36411374 10.1038/s41565-022-01252-8

[7] Osterhoudt, G. B. et al. Colossal mid-infrared bulk photovoltaic effect in a type-I Weyl semimetal. *Nat. Mater.* **18**, 471–475 10.1038/s41563-019-0297-4 (2019). 30833781 10.1038/s41563-019-0297-4

[8] Wang, Z. et al. Giant infrared bulk photovoltaic effect in tellurene for broad-spectrum neuromodulation. *Light Sci. Appl.* **13**, 277 10.1038/s41377-024-01640-w (2024). 39327457 10.1038/s41377-024-01640-w PMC11427709

[9] Peng, M. et al. Blackbody-sensitive room-temperature infrared photodetectors based on low-dimensional tellurium grown by chemical vapor deposition. *Sci. Adv.* **7**, eabf7358 10.1126/sciadv.abf7358 (2021). 33863732 10.1126/sciadv.abf7358 PMC8051875

[10] Tong, L. et al. Stable mid-infrared polarization imaging based on quasi-2D tellurium at room temperature. *Nat. Commun.* **11** 2308 10.1038/s41467-020-16125-8 (2020). 32385242 10.1038/s41467-020-16125-8 PMC7210936

[11] Chynoweth, A. G. Surface space-charge layers in barium titanate. *Phys. Rev.* **102**, 705–714 10.1103/PhysRev.102.705 (1956).

[12] Ichiki, M. et al. Photovoltaic effect of lead lanthanum zirconate titanate in a layered film structure design. *Appl. Phys. Lett.* **84**, 395–397 10.1063/1.1641528 (2004).

[13] Choi, T. et al. Switchable ferroelectric diode and photovoltaic effect in BiFeO_3_. *Science* **324**, 63–66 10.1126/science.1168636 (2009). 19228998 10.1126/science.1168636

[14] Grinberg, I. et al. Perovskite oxides for visible-light-absorbing ferroelectric and photovoltaic materials. *Nature* **503**, 509–512 10.1038/nature12622 (2013). 24213630 10.1038/nature12622

[15] Xiao, Z. G. et al. Giant switchable photovoltaic effect in organometal trihalide perovskite devices. *Nat. Mater.* **14**, 193–198 10.1038/nmat4150 (2015). 25485985 10.1038/nmat4150

[16] Quattropani, A. et al. Tuning photovoltaic response in Bi_2_FeCrO_6_ films by ferroelectric poling. *Nanoscale* **10**, 13761–13766 10.1039/C8NR03137A (2018). 29993081 10.1039/c8nr03137a

[17] Xu, S. Y. et al. Electrically switchable berry curvature dipole in the monolayer topological insulator WTe_2_. *Nat. Phys.* **14**, 900–906 10.1038/s41567-018-0189-6 (2018).

[18] Zhang, Y. J. et al. Enhanced intrinsic photovoltaic effect in tungsten disulfide nanotubes. *Nature* **570**, 349–353 10.1038/s41586-019-1303-3 (2019). 31217597 10.1038/s41586-019-1303-3

[19] Li, Y. et al. Enhanced bulk photovoltaic effect in two-dimensional ferroelectric CuInP_2_S_6_. *Nat. Commun.* **12**, 5896 10.1038/s41467-021-26200-3 (2021). 34625541 10.1038/s41467-021-26200-3 PMC8501070

[20] Jiang, J. et al. Flexo-photovoltaic effect in MoS_2_. *Nat. Nanotechnol.* **16**, 894–901 10.1038/s41565-021-00919-y (2021). 34140672 10.1038/s41565-021-00919-y

[21] Ma, C. et al. Intelligent infrared sensing enabled by tunable moiré quantum geometry. *Nature* **604**, 266–272 10.1038/s41586-022-04548-w (2022). 35418636 10.1038/s41586-022-04548-w

[22] Rees, D. et al. Helicity-dependent photocurrents in the chiral Weyl semimetal RhSi. *Sci. Adv.* **6**, eaba0509 10.1126/sciadv.aba0509 (2020). 32832618 10.1126/sciadv.aba0509 PMC7439497

